# Diabetes Mellitus Promotes the Development of Atherosclerosis: The Role of NLRP3

**DOI:** 10.3389/fimmu.2022.900254

**Published:** 2022-06-29

**Authors:** Jingxue Ye, Lanfang Li, Min Wang, Qiuxiao Ma, Yu Tian, Qiong Zhang, Jiushi Liu, Bin Li, Bengang Zhang, Haitao Liu, Guibo Sun

**Affiliations:** ^1^ Institute of Medicinal Plant Development, Chinese Academy of Medical Sciences and Peking Union Medical College, Beijing, China; ^2^ Xiyuan Hospital, China Academy of Chinese Medical Sciences, Beijing, China

**Keywords:** atherosclerosis, diabetes mellitus, NLRP3, endothelial dysfunction, hyperglycemia, dyslipidemia, NLRP3 inhibitor

## Abstract

Atherosclerosis is one of the main complications of diabetes mellitus, involving a variety of pathogenic factors. Endothelial dysfunction, inflammation, and oxidative stress are hallmarks of diabetes mellitus and atherosclerosis. Although the ability of diabetes to promote atherosclerosis has been demonstrated, a deeper understanding of the underlying biological mechanisms is critical to identifying new targets. NLRP3 plays an important role in both diabetes and atherosclerosis. While the diversity of its activation modes is one of the underlying causes of complex effects in the progression of diabetes and atherosclerosis, it also provides many new insights for targeted interventions in metabolic diseases.

## Introduction

Diabetes mellitus is a major and growing problem worldwide, not only in the so-called developed countries (1, 2). According to a recent report from the International Diabetes Federation, about 537 million adults (aged 20-79) worldwide will have diabetes in 2021 (1 in 10 people will have diabetes). That number is expected to rise to 643 million by 2030.That will rise to 783 million by 2045. During this period, the world’s population is estimated to have grown by 20 percent, while the number of people living with diabetes is estimated to have increased by 46 percent. DM is divided into two types, namely, type 1 diabetes mellitus (T1DM), which involves the destruction of beta cells in the islets, leading to an absolute lack of insulin secretion, and type 2 diabetes mellitus (T2DM), which involves an insufficient insulin secretion or combined insulin resistance (3) (4). In addition to nephropathy and microvascular disease, cardiovascular disease frequently occurs in patients with T1DM and T2DM, and is the main cause of morbidity and death, accounting for more than 80% of deaths among those with DM, and otherwise significantly reducing their quality of life (5). T2DM is often accompanied by dyslipidemia (6). Elevated glucose levels have been identified as an independent predictor of platelet-dependent thrombosis in patients with coronary artery disease (7). Poor glycemic control adversely affects the large blood vessels, accelerating atherosclerosis and cardiovascular disease, manifesting as myocardial infarction, stroke, and peripheral artery disease (8, 9).

Atherosclerosis, one of the main complications of DM, involves many pathogenic factors, such as endothelial dysfunction, oxidative stress and so on (10, 11). Hyperglycemia and hyperlipidemia are related factors for the accelerated development of atherosclerosis (12). Recent evidence suggests that dysglycemia and dyslipidemia can induce endothelial cell dysfunction (13). Moreover, oxidative stress is the main cause of insulin resistance in T2DM patients, and it can also promote the oxidation of low-density lipoprotein (LDL) (4). The coexistence of high glucose levels and persistent oxidative stress leads to the formation of advanced glycation end products (AGEs). AGEs, through their receptor interactions (RAGEs), play an important role in the vascular complications of diabetes (14). AGEs can upregulate inflammatory signaling pathways and induce reactive oxygen species production and apoptosis. AGES can also exert their pathogenic effects by modifying additional or intracellular proteins and activating cellular signaling cascades through RAGEs (15). Glycosylation of extracellular proteins can directly contribute to atherosclerosis. Furthermore, AGE/RAGE signaling can promote increased hemagglutinin-like oxidized low-density lipoprotein (ox-LDL) receptor (LOX-1) expression in endothelial cells, thereby promoting ox-LDL uptake.

Diabetes is associated with accelerated atherosclerosis, leading to widespread vascular disease (16). Chronic hyperglycemia (17), dyslipidemia (18), insulin resistance (19), and glucose/lipid oxidation end products (1) are typical risk factors for diabetes and may lead to vascular complications. Glucose control, lipid-lowering agents, antioxidants and anti-inflammatory agents have shown some degree of efficacy in reducing the progression of diabetes-related atherosclerosis. However, more research is needed to identify specific targets based on novel mechanisms for the treatment and prevention of diabetes-associated atherosclerotic cardiovascular disease.

## NLRP3

**Figure 1:**
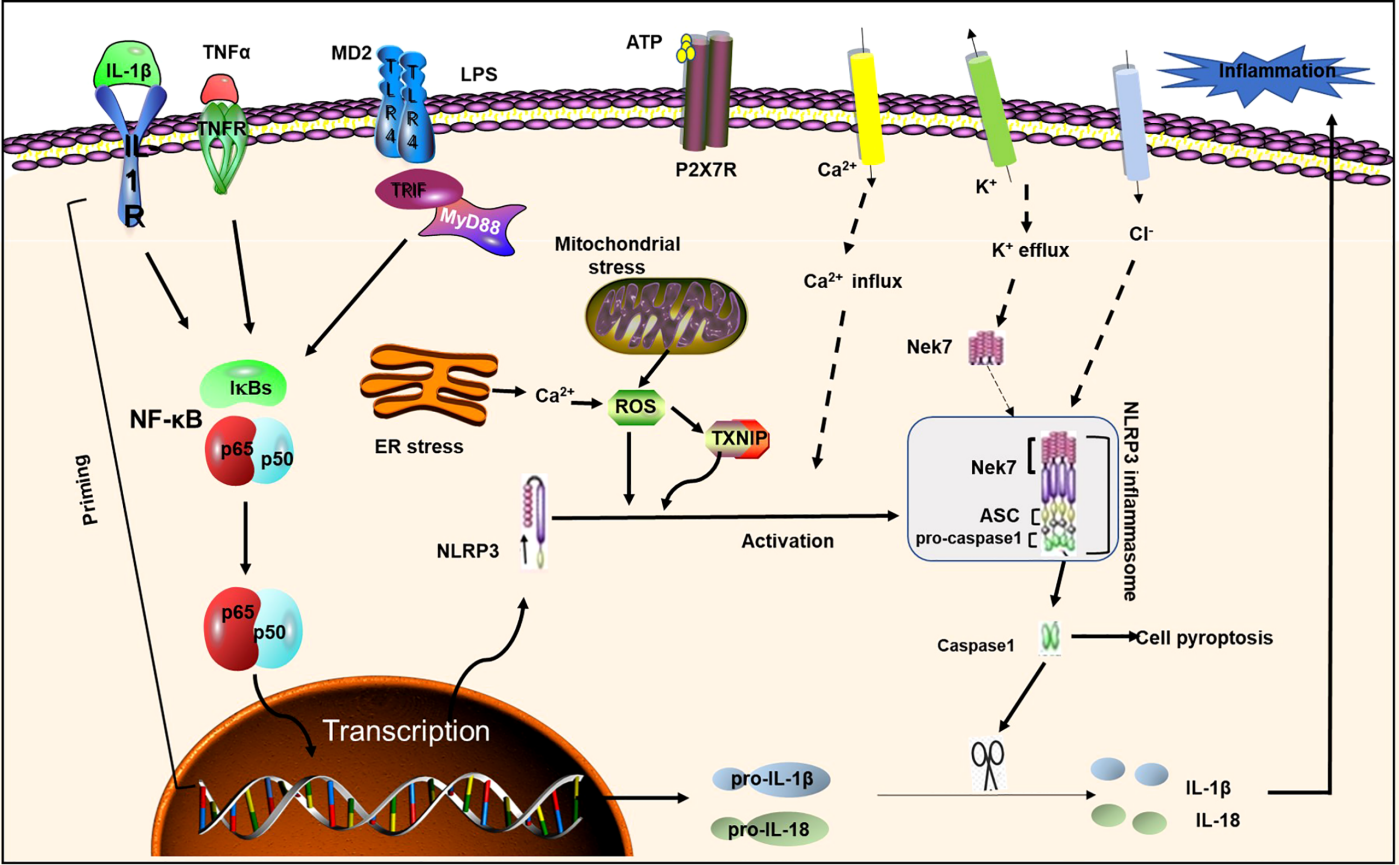
NLRP3 inflammasome priming and activation. NLRP3 inflammasome activation requires two signals: Signal 1 (initiation) is provided by activating cytokines or PAMPs, resulting in transcriptional upregulation of normative and non-standard NLRP3 inflammasome components. Signal 2 (activation) is activated by K ^+^ efflux, Ca ^2+^ influx, and mitochondrial reactive oxygen species, etc. ASC; IL-1R1, IL-1 receptor type1; NEK7, NIMA-related kinase7; NF-κB, nuclear factor-κB; P2X7, P2X purinoceptor7; ROS, reactive oxygen species; TLR, toll-like receptor; TNF α, tumor necrosis factor α; TNFR, tumor necrosis factor receptor;TXNIP, thioredoxin-interacting protein; MD2, myeloid differentiation factor 2;LPS, lipopolysaccharide; ER Stress, endoplasmic reticulum; TIRF, toll-interleukin 1 receptor (TIR) -inducing interferon; MyD88, myeloid differentiation primary response gene 88.

Inflammation underlies a wide range of physiological and pathological processes. Inflammasomes are polymorphic complexes formed by pattern recognition receptors activated by various physiological or pathogenic stimuli that create an innate immune response with the ability to clear pathogens and damaged cells (20). The most well-known inflammasome is NLRP3, which belongs to the family of nucleotide-binding and oligomerization domain-like receptors (NLRs), also known as “pyrin domain-containing protein 3” (21). The NLRP3 inflammasome multiprotein complex is composed of sensor-NLRP3, adaptor of the caspase recruitment domain-apoptosis-associated spotted protein, and effector protein -caspase 1. The 3 domains are interacted to promote caspase cleavage and the subsequent maturation and secretion of interleukins IL-18 and IL-1β (22) (**Figure 1**). ASC contains an N-terminal pyrin domain and a C-terminal caspase recruitment domain (23). NLRP3 contains three domains: C-terminal leucine-rich repeats, a central nucleotide domain called the NACHT domain, and an N-terminal effector domain. Caspase-1 has conservative domains for homophilic interaction, and it also contains CARD and catalytic domains (24). Multiple studies have shown that NLRP3 inflammasome, IL-1β, IL-18 and pyroptosis have a decisive and important role in various diseases, such as DM, atherosclerosis, and Alzheimer’s disease (25). Activation of the NLRP3 inflammasome requires the completion of two signaling steps. The first signal, including lipopolysaccharide or cytokines, can activate the NF-κB pathway and up-regulate the expression of NLRP3 and IL-1β. The second signal including a variety of pathogen-associated molecular patterns (PAMPs) and damage-associated molecular patterns (DAMPs), such as K^+^ efflux, ROS increase, endoplasmic reticulum stress, mitochondrial dysfunction, Ca^2+^ signaling, and lysosomal disruption, can promote the assembly of the NLRP3 inflammasome complex, so that pro-caspase-1 is cleaved into active caspase-1, which converts pro-IL-1β, pro-IL -18 cleaves to the mature form and causes pyroptosis (26, 27). NLRP3 plays a key role in inflammatory responses and various diseases. Growing evidence suggests that the NLRP3 inflammasome contributes to the development of DM and atherosclerosis, and that inactivation of NLRP3 inflammation is beneficial for the treatment of these diseases (28) (29). We next briefly introduce the role of NLRP3 in DM and atherosclerosis.

### DM and NLRP3

DM is a chronic inflammatory metabolic disorder caused by a variety of etiologies, which is related to the release of tumor necrosis factor (TNF) and lipokinase from adipose tissue (22). Il-1 β is a key inflammatory mediator during the pathogenesis of DM, which can promote insulin resistance, disrupt the function of islet β cells, and lead to islet cell apoptosis (30). The overexpression of pro-inflammatory factors may further induce DM complications (31). In recent years, multiple studies have suggested that NLRP3 inflammasome is related to DM and its complications (32). For example, Tseng et al. demonstrated that hyperglycemia high glucose induces activation of NLRP3-ASC inflammasome, leading to casparase-1 activation and secretion of IL-1β and IL-18 in human monocyte cell lines (33). Wang et al. demonstrated that NLRP3 gene polymorphism is closely associated with type 2 diabetes susceptibility (34). Lee et al. also reported upregulation of NLRP3 and its downstream molecules in T2DM and atherosclerosis (35). Söderbom G et al. identified the NLRP3 inflammasome as a bridge between neuro-inflammation in metabolic and neurodegenerative diseases (36). Thus, activation of NLRP3 appears to be a major mechanism in T2DMand its complications.

Insulin resistance and pancreatic β-cell secretion dysfunction in patients with type 2 diabetes can worsen glycemic control, so it is important to delay and control the progressive decline of pancreatic β-cell function in diabetic patients (37). NLRP3 activation and IL-1β stimulation affect islet function and insulin secretion and are key triggers of islet damage (38). Persistent hyperglycemia in pancreatic islets induces ROS accumulation, leading to elevated thioredoxin-interacting protein (TXNIP), activation of the NLRP3 inflammasome, and induction of caspase-1-dependent IL-1β maturation. Youm et al. also demonstrated that reducing NLRP3 inflammasome-dependent IL-1β production reduced islet fibrosis in a mouse model of obesity (39). Furthermore, NLRP3 can affect T cell activation and maturation. The elimination of NLRP3 alters T cell migration to the islet, a key pathogenic process leading to β cell damage (40). In addition, NLRP3 knockout down-regulated the level of chemokines CCL5 (C-C motif ligand 5) and CXCL10 (C-X-C motif ligand 10) in islet cells, suggesting that NLRP3 regulates chemotaxis. It has been demonstrated that T2DM is associated with obesity (41). Most patients with T2DM have visceral obesity, which is related to insulin resistance (37). The NLRP3 inflammasome is critical in metabolic dysregulation and control of obesity-related IR and pancreatic β-cell dysfunction (23). During obesity, macrophages in adipose tissue express high levels of NLRP3 and release inflammatory cytokines such as TNF-α and IL-1β. High levels of IL-1β may make obese patients less sensitive to insulin (42).

**Figure 2:**
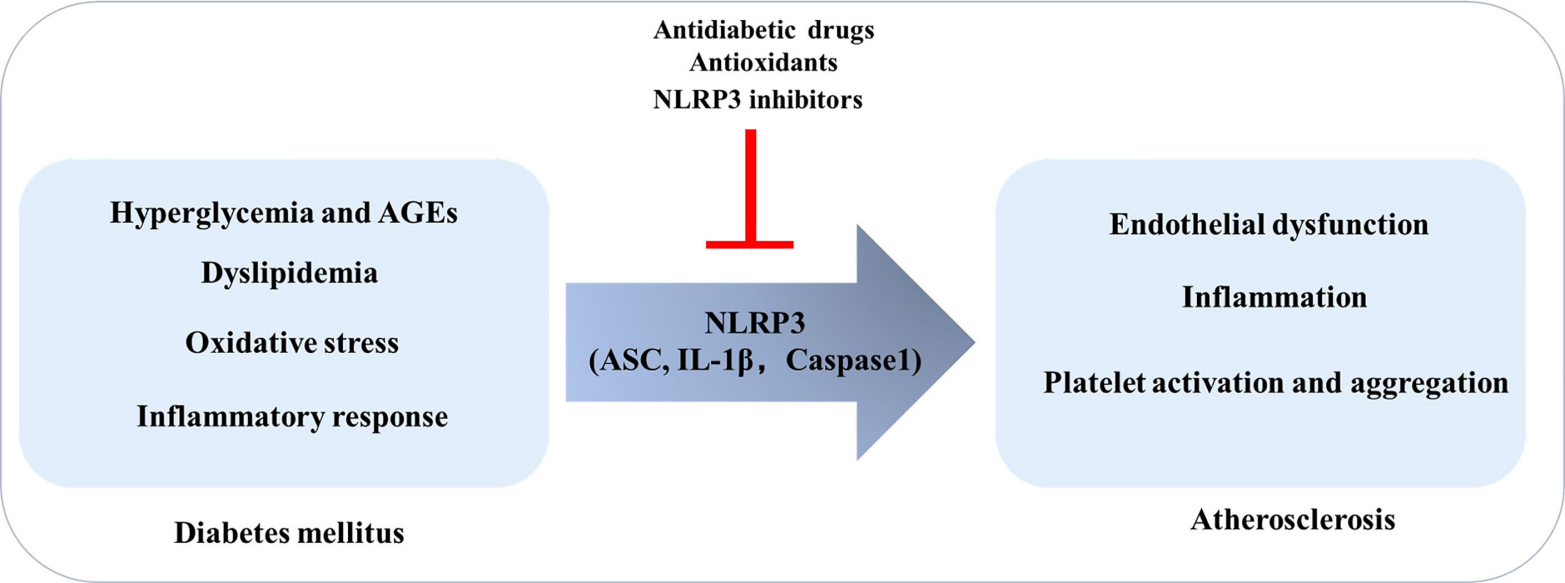
Diabetes Mellitus promotes the development of atherosclerosis: the role of NLRP3. In the context of diabetes mellitus, risk factors such as high glucose, oxidative stress, and inflammation can induce endothelial dysfunction, inflammation, and platelet activation and aggregation by regulating NLRP3 inflammasome, thereby promoting atherosclerosis.

Therefore, it can be concluded that the factors that promote the development of T2DM may induce or exacerbate the disease by regulating NLRP3 and signaling pathways, while the inhibition of NLRP3 inflammasome and its related molecules provides the possibility for the treatment of DM and its complications (**Figure 2**).

### Atherosclerosis and NLRP3

Atherosclerosis is a chronic inflammatory disease in which lipids deposit in areas where blood flow in the large arteries is disturbed, leading to the formation of plaques. Plaque rupture or erosion can lead to acute cardiovascular events, such as heart attack and stroke (43). Atherosclerosis is also a chronic inflammatory disease. Inflammation is involved in various processes of atherosclerosis formation, including the formation of lipid streaks, the formation of fibrous plaques and the stability of plaques (44). Elevated NLRP3 inflammasome expression levels have been found in the aortas of patients with coronary atherosclerosis (45). Studies have shown that the protein levels of NLRP3 inflammasome, ASC, caspase-1, IL-1β, and IL-18 are significantly higher in plaques than in healthy arteries, and that the plaque-unstable type is more prevalent than the plaque-stable type (29).

In 2010, Duewell et al. showed for the first time the important role of the NLRP3 inflammasome in the progression of atherosclerosis (46). They used LDL receptor-deficient (LDLR^-/-^) mice with NLRP3 ^-/-^, ASC ^-/-^ and IL-1α/β^-/-^ to show that knockout NLRP3, ASC and IL-1α/β protected mice and reduced atherosclerotic lesions (46). Moreover, accumulating evidence has shown that NLRP3 activation products IL-1β and IL-18 both play an important role in the occurrence of atherosclerosis. Ox-LDL is the most important risk factor for atherosclerosis (47). Studies have reported that ox-LDL can induce macrophage secretion of IL-1β by activating the NLRP3 inflammasome. Ox-LDL-induced NLRP3 inflammasome activation is dependent on ROS production (48). Huang et al. found that exposure to ox-LDL in endothelial cells or feeding mice with a high-fat diet triggered ROS production, which promoted NLRP3 inflammasome activation and IL-1β secretion (49). In contrast, endothelial-specific NLRP3 knockout attenuated the severity of atherosclerosis in high-fat diet mice. Furthermore, acute hypercholesterolemia exacerbated endothelial dysfunction in, which was significantly ameliorated by inhibit NLRP3 (50).

Several studies have also shown that the NLRP3 inflammasome promotes to the development of atherosclerosis by influencing several pathogenic events, such as oxidative stress, mitochondrial dysfunction, endoplasmic reticulum stress, and lysosomal disruption (51). Owing to its critical role in the development of atherosclerosis, NLRP3 is a promising therapeutic target for atherosclerosis. A broader understanding of inflammasome biology and activation or inhibition mechanisms is needed to determine the value of these complexes as potential therapeutic targets in atherosclerosis.

## Mechanism of NLRP3 in the Process of DM-Promoting Atherosclerosis

Studies on individuals with DM have shown an increased risk and accelerated development of atherosclerosis (10, 52). DM and atherosclerosis are linked through several pathological pathways, such as endothelial dysfunction, dyslipidemia, and oxidative stress (53). NLRP3 acts as a sensor of metabolic stress, linking metabolic disturbances to inflammation (54). We next discuss the specific mechanism of action of NLRP3 in DM-promoting atherosclerosis.

### Hyperglycemia

In general, hyperglycemia-induced pathological changes are the cause of complications in patients with DM. High blood sugar levels and the persistence of oxidative stress would lead to AGEs (55). AGEs produce their disease-causing effects by modifying extracellular or intracellular proteins and activating cellular signaling cascades through RAGE (56). Glycosylation of extracellular proteins directly contributes to atherosclerosis. Changes in LDL molecules prevent them from being absorbed by receptors, leading to phagocytosis by monocytes and macrophages, resulting in foam cells (57). In addition, glycosylated fibrinogen becomes resistant to proteolysis, leading to a tendency toward thrombosis (58).

Glucose, as DAMPs, is the first signal of upregulation of NLRP3 and pro-IL-1β. High glucose stimulation can induce macrophages to polarize into an M1 phenotype, partly through the NLRP3/IL-1 β pathway, which may be one of the mechanisms leading to diabetic complications (59). Wan et al. demonstrated that NLRP3 knockdown inhibited the expression of adhesion molecules ICAM-1 and VCAM-1 in intima, reduced atherosclerosis and stabilized atherosclerotic plaques.*In vitro*, high glucose in HUVECs enhances the expression of NLRP3 inflammasome components and secretion of IL-1β.In addition, high glucose or IL-1β promotes the expression of adhesion molecules that are inhibited by NLRP3 knockdown or IL-1β receptor antagonists (60). Hyperglycemia induces activation of TLR4, leading to NF-κB promotion. This process further activates the NLRP3 inflammasome, leading to increased expression of pro-inflammatory cytokines (61). Finally, inflammation and cell death will be significantly increased. TXNIP is a central regulator of glucose and lipid metabolism. Hyperglycemia can also induce TXNIP overexpression (62). Glucose also affects monocyte/macrophage activation, and monocytes grown under high glucose conditions show increased expression of cytokines, IL-1β, and IL-6.

Oxidative stress accelerates the progression of atherosclerosis by inducing inflammation, endothelial dysfunction, thrombosis, and plaque instability (63). In diabetic patients, multiple pathways induce oxidative stress, including glucose oxidation, enhanced glycosylation, activation of the AGE-RAGE axis, and enhanced polyol pathway (64). Persistent hyperglycemia, with increased glycolysis, can lead to mitochondrial dysfunction, which induce overproduction of mitochondrial ROS (mt-ROS), which in turn activates the NLRP3 inflammasome (61). High glucose levels have been shown to induce ROS production in endothelial cells by triggering the Ca^2+^ and ERK1/2 pathways (65). When blood sugar levels are high, glycolysis is enhanced, which increases the production of superoxide in cells.

ROS acts as an intermediate trigger for NLRP3 inflammasome activation, exacerbating the ensuing inflammatory cascade and leading to cellular damage (66). ROS inhibitors prevented high glucose-induced caspase-1 activation and IL-1β production in endothelial cells, suggesting that high glucose levels induce endothelial NLRP3 inflammasome production through ROS production. Excessive ROS produced during hyperglycemia stimulates vascular cells to secrete IL-1β, which disrupt tight junctions, increase permeability, and allow vascular permeability leaking fluids and molecules into the inner membrane, such as ox-LDL. Summary, ROS link the interaction between the NLRP3 inflammasome and endothelial dysfunction.

Hyperglycemia-induced endothelial oxidative stress is associated with the development of diabetic complications, in part by inducing endothelial cell apoptosis and promoting endothelial activation and vascular inflammation (67). Nuclear factor redline 2 associated factor-2 (Nrf2) is a transcription factor activated by oxidative stress that regulates the expression of ROS detoxification and antioxidant genes (68). Zoltan et al. elucidate the homeostasis role of adaptively induced NRF2-driven free radical detoxification mechanisms in endothelial protection under diabetes (69). Nrf2 can be pharmacologically activated by polyphenol resveratrol or sulforaphane, which leads to a significant induction of the cellular antioxidant system, increasing GSH levels and correspondingly reducing oxidative stress. Importantly, resveratrol, H2S, and chlorogenic acid can effectively attenuate vascular ROS production and improve endothelial function and/or attenuate hyperglycaemic induced endothelial oxidative stress in diabetic animal models (70–72). Therefore, pharmacological treatment to promote the induction of Nrf2 driven homeostasis pathway could significantly promote intervention strategies to prevent vascular disease in diabetic patients.

These findings suggest that hyperglycemia directly or indirectly stimulates the activation of NLRP3 inflammasome in different ways, thereby inducing immune response and inflammation, and ultimately promoting the occurrence of diabetic vascular complications.

### Dyslipidemia

DM is closely related to dyslipidemia, and the two affect each other as the common soil of arteriosclerotic diseases (73). T2DM is often associated with lipid abnormalities, manifested by increased very-LDL (VLDL) and LDL-cholesterol (LDL-C), and decreased high-density lipoprotein cholesterol (HDL-C) (74). Therefore, monitoring biochemical parameters such as blood lipids and blood glucose status in patients with DM and their relationship with dyslipidemia can reduce the prevalence of diabetic cardiovascular disease.

Dyslipidemia has been found to induce inflammation through pathways including NLRP3 activation. Cholesterol is a common participant in dyslipidemia. Kristiina et al. found that cholesterol crystals activate NLRP3 by suggesting mechanisms involving potassium efflux and leakage of cathepsin B into the cytoplasm (75). Duewell and colleagues found that cholesterol crystals activate the NLRP3 inflammasome in mouse macrophages through lysosomal damage and cathepsin-mediated mechanisms (46). In atherosclerosis, inflammasome-mediated IL-1β release promotes an inflammatory environment that promotes disease progression. Cholesterol crystals are phagocytosed by macrophages, leading to lysosomal instability, and K^+^ efflux, which then activates the NLRP3 inflammasome (76). Likewise, CANTOS trial showed that cholesterol has been identified as inducing NLRP3 inflammasome activation and atherogenesis (77). ATP-binding cassette transporter A1 and G1 (ABCA1/G1) have been found to be transmembrane proteins that mediate cholesterol efflux to apolipoprotein A and HDL-C, and then inhibit NLRP3 inflammasome activation. Therefore, ABCA1/G1 has a protective effect on the activation of the NLRP3 inflammasome (77). Thus, activation of inflammatory bodies induced by cholesterol crystals may represent an important link between cholesterol metabolism and inflammation in atherosclerotic lesions.

Similarly, Wang et al. and Li et al. found that OX-LDL could induce cell damage through NLRP3-mediated pyroapoptosis (78, 79). Zhang et al. found that metformin can reduce NLRP3 inflammatory body activity in ox-LDL-stimulated macrophages, thereby reducing pro-inflammatory and pro-atherogenic responses in ox-LDL-stimulated macrophages (80). Qian et al. demonstrated that astragaloside IV protects endothelial progenitor cells from ox-LDL damage through the NLRP3 inflammasome pathway (81). Stimulation with ox-LDL in THP-1 macrophages upregulated NLRP3, and IL-1β (82). Other lipids, such as SFAs (palmitate and stearate) and their metabolites ceramides and triglycerides, are also risk factors for atherosclerosis, and they can also trigger NLRP3 inflammasome-dependent pyroptosis in macrophages (83).

Diabetes, often accompanied by hyperlipidemia, is a risk factor for atherosclerosis. Risk factors in hyperlipidemia lead to activation of NLRP3 inflammasome in endothelial cells. Early hyperlipidemia promotes monocyte recruitment through caspase-1 and ROS production, followed by endothelial cell apoptosis. Therefore, inhibition of NLRP3 dependent caspase-1 production may alleviate the development of atherosclerosis. Hyperlipidemia also includes elevated level of LDL. LDL can trigger endoplasmic reticulum stress and ROS generation, which then activates the NLRP3 inflammasome and ultimately leads to endothelial cell damage (84). Furthermore, hyperglycemia induce upregulation of E74-like ETS transcription factor 3 (ELF3), leading to NLRP3 inflammasome activation (85). Dyslipidemia and chronic inflammation are the main drivers of plaque formation leading to diabetic atherosclerosis. Therefore, real-time monitoring of lipid levels and blood glucose status can reduce the diabetic cardiovascular disease induced DM.

### Endothelial Dysfunction

Endothelial dysfunction occurs in the early stages of cardiovascular disease and is one of the first manifestations of T2DM and cardiovascular disease (86). Endothelial barrier dysfunction is characterized by the abnormal secretion of various inflammatory mediators, including IL-1β, TNFα, histamine, and bradykinin, leading to disruption of inter-endothelial junctions (87). The factors that contribute to endothelial dysfunction in DM include inflammation, dyslipidemia, hyperglycemia, and oxidative stress. Accumulating studies have shown that the NLRP3 inflammasome also plays an important role in endothelial dysfunction (88). Activation of NLRP3 inflammasome is the cause of vascular endothelial dysfunction in diabetes mellitus (89).

Endothelial cell damage is the first step in atherosclerosis, and both mechanical damage (e.g., hemodynamics) and chemical damage (e.g., from smoking, hyperlipidemia, and hyperglycemia) can lead to endothelial cell damage (90). In endothelial cells, these stimuli trigger NLRP3 inflammasome activation, which initiates the cells to secrete IL-1β and IL-18, promoting further endothelial inflammatory processes. Secreted IL-1β and IL-18 also promote the secretion of adhesion molecules and secondary inflammatory factors, such as E-selectin, ICAM-1, VCAM-1, IL-6, inducing recruitment of inflammatory cells. In addition, the production of AGEs is also critical during endothelial dysfunction. The interaction between AGEs and RAGE induces oxidative stress and inflammatory responses in endothelial cells, which may lead to endothelial dysfunction (91). Endothelial barrier disruption caused by excess circulating free fatty acids in obese individuals appears to be mediated by inflammasome activation. Vascular calcification, which is a general feature in patients with atherosclerosis and T2DM, is also associated with NLRP3 inflammasome activation.

The endothelium forms an interface and acts as a semi-permeable barrier. Enhanced endothelial permeability leads to endothelial dysfunction. Inflammatory mediators can activate various signaling pathways, leading to increased endothelial permeability. Studies have shown that exposure of endothelial cells to hyperglycemia, accompanied by changes in their secretion, increases endothelial cell permeability. High glucose concentrations also promote more glycation of LDL in patients with DM and, with endothelial dysfunction, glycated LDL promotes increased expression of adhesion molecules. Lian et al. have shown that high glucose levels in the coronary endothelial cells of streptozotocin diabetic mice were found to induce the release of high mobility group box 1 (HMGB1) through the aggregation and activation of the NLRP3 inflammasome, and that, in endothelial monolayer cells, upregulation of tight junction proteins ZO-1/ZO-2 disrupts endothelial tight junctions (92). The aggregation and activation of the NLRP3 inflammasome is mediated by the ROS signaling pathway. Blockade of NLRP3 inflammasome activation by ROS inhibitors significantly down-regulated ZO-1/ZO-2 and alleviated endothelial hyperpermeability in DM, suggesting a loss of function of the NLRP3 inflammasome (92). It can prevent the destruction of tight junctions under high glucose conditions and improve endothelial function. A comprehensive understanding of the molecular mechanisms of inflammation associated with endothelial dysfunction will contribute to the prevention and control of diabetes induced inflammation.

### Inflammation

Atherosclerosis is a chronic inflammatory disease, and the link between T2DM and inflammation has been established, with evidence of chronic inflammation being present in both those with DM and with IR. In patients with DM and atherosclerosis, substances that induce inflammation include AGEs, cholesterol, and uric acid (93). The development of atherosclerosis in DM has been reported to involve inappropriate persistent inflammation induced by PAMPs and DAMPs overactivation of the NLRP3 inflammasome.

In DM, vascular endothelium actively participates in the regulation of inflammatory progression and plays an important role in cardiovascular homeostasis as a dynamic adaptive interface. Proinflammatory cytokines such as TNF-α, IL-1β and IL-6 play an important role in the development of endothelial cell injury. TNF-α induced cytotoxicity, IL-6 increased endothelial permeability, IL-1β induced NO synthase expression, and synergistic effect with TNF-α (94). Studies have shown that mice lacking NLRP3 inflammasome components exhibit anti-inflammatory and anti-atherosclerotic phenotypes (95). These mice were also protected from insulin resistance and metabolic dysfunction associated with obesity-induced DM, suggesting that NLRP3 inflammasome may act as a transducer to detect danger signals and trigger inflammatory responses in these diseases.

### Platelet Activation and Aggregation

The inflammatory state that occurs in DM promotes platelet hyperresponsiveness and adherence to the endothelium, resulting in a thrombotic phenotype and increased cardiovascular mortality (96, 97). Therefore, studies aimed at addressing platelet hyperreactivity is of high clinical value for combating DM-related atherosclerosis. At the platelet level, DM can enhance platelet susceptibility to activation through a variety of mechanisms. Multiple mechanisms caused by metabolic and cellular abnormalities have been suggested to increase platelet reactivity in diabetic patients. Among them, hyperglycemia increases platelet reactivity by glycosylating platelet surface proteins, activating protein kinase C, inducing p-selectin expression and osmosis, which promote platelet activation and adhesion (98). Metabolic diseases often associated with diabetes may themselves play a role in platelet hyperresponsiveness, including obesity, dyslipidemia, and increased systemic inflammation (99). Obesity also leads to platelet dysfunction, mainly in adhesion and activation, through mechanisms including increased cytoplasmic calcium concentration and enhanced oxidative stress (100). Dyslipidemia, especially hypertriglyceridemia, also affect platelet reactivity through different mechanisms (101). Endothelial dysfunction, another hallmark of diabetes, enhances platelet reactivity by reducing NO and PGI2 production and promotes the prethrombotic state by increasing TF production (7, 102). This increases the osmotic pressure and enhances the platelet responsiveness to stimulation. Accumulating evidence has shown that TLRs and NLRP3 are the two major components in promoting platelet activation and aggregation. Specifically, TLR4/NLRP3 pathway mediated inflammation significantly increases the release of various inflammatory and adhesion factors that promote platelet aggregation and adhesion (103). NLRP3 is upregulated in platelets, which, *via* HMGB1 mediation, promotes platelet activation, increased aggregation, increased microvascular thrombosis and further exacerbates disease (104). The abnormal increase of inflammatory factors in the blood of patients with DM can further promote thrombosis. In particular, the NLRP3 inflammasome-mediated secretion of IL-1β and IL-18 promotes platelet aggregation (105), which is critical for the thrombotic phenotype in DM. Studies have shown that tetramethylpyrazine alleviates hyper-platelet responses and endothelial adhesion in a DM-induced prothrombotic phenotype by regulating the NLRP3 inflammasome (106). Therefore, modulating the NLRP3 related inflammatory in DM to prevent platelet activation and adhesion to endothelial cells may be considered a promising strategy for reducing the risk of cardiovascular events in patients with DM.

## Drug Treatment

In recent years, antidiabetic drugs have been found to reduce endothelial disease by blocking NLRP3 inflammasome activation, which provides a possibility for the treatment of diabetic atherosclerotic lesions. In general, antidiabetic drugs reduce ox-LDL uptake by vascular cells and subsequent inflammatory signaling, thereby preventing macrophage adhesion and infiltration. Therefore, the anti-inflammatory, antioxidant, and anti-apoptotic properties of antidiabetic drugs can eliminate the changes caused by ox-LDL, which is very beneficial for the control of atherosclerosis in patients with DM. The regulatory mechanisms of these drugs on NLRP3 are summarized as follows.

### Antidiabetic Drugs

Metformin is a classic antidiabetic drug with continuous reports of its cardiovascular protective effects. Tang et al. found that metformin could inhibit high glucose-induced NLRP3 inflammasome activation and attenuate DM-accelerated atherosclerosis, which worked through the AMPK signaling pathway (107).. Li et al. found that metformin can inhibit high glucose-induced changes in Trx1/TXNIP, while reducing intracellular ROS and inhibiting the NLRP3 inflammasome, which significantly blocked NLRP3 and macrophage markers in atherosclerotic lesions in diabetic mice (108). This suggests that metformin can also inhibit NLRP3 inflammasome-mediated macrophage recruitment in atherosclerotic lesions in diabetic mice. Research shows that vascular normalization is linked positively with hampered NLRP3 inflammasome activation (109). Metformin has significant vascular protection, mainly through down-regulation of HIF1α and regulation of PDGF-B, thus promoting the normalization of dysfunctional vessels (110–114). Interestingly, metformin has the potential to inhibit NLRP3 inflammasome activity in chronic diseases, such as T2DM, while promoting inflammasome activity in acute diseases, such as bacterial infections (115, 116).

Dipeptidyl peptidase 4 (DPP-4) inhibitors are a class of potent hypoglycemic agents for the treatment of T2DM. DPP-4 inhibitors have also shown beneficial effects on oxidative stress, and endothelial function in patients with T2DM, and to exercise an anti-atherosclerosis function. For example, sitagliptin significantly reduced ox-LDL-induced expression of NLRP3, TLR4 and IL-1β in THP-1 cells (117). Qi et al. found that vildagliptin protected mitochondrial function and restored endothelial function by inhibiting the NLRP3-HMGB1 pathway level of NO synthase (118), suggesting its protective effect against endothelial dysfunction.

Dulaglutide is a glucagon-like peptide-1 receptor agonist (GLP-1 RA) approved for the treatment of T2DM. GLP-1R agonists have also been reported to exhibit cardiovascular benefits, with potential antiatherosclerosis effects. Recently, dulaglutide has been shown to protect against high glucose-induced endothelial dysfunction through inhibition of NLRP3 inflammasome activation and inhibition of NOX4-ROS-TXNIP-NLRP3 signaling (119).

Acarbose, an alpha-glucosidase inhibitor, is a postprandial-acting antidiabetic drug. Acarbose is protective against endothelial dysfunction by inhibiting ROS production, which helps block the activation of the NLRP3 inflammasome (120). In addition, following the use of acarbose, decreased expression of the NLRP3 inflammasome, improving the vascular hyperpermeability, which was attributed to enhanced expression of the connexin ZO-1 and VE-cadherin (120).

Fenofibrate, a selective agonist of the peroxisome proliferator-activated receptor alpha, prevents progression of microvascular complications in T2DM. Deng et al. found that fenofibrate attenuated endothelial cell dysfunction, which due to inhibition of the NLRP3 inflammasome pathway (121).

Cilostazol (a phosphodiesterase 3 inhibitor) is an antiplatelet agent that also dilates arterial blood vessels. It can alleviate adverse effects on blood vessels in patients with DM and significantly reduce free fatty acid-induced activation of the NLRP3 inflammasome in endothelial cells. Cilostazol also protects the function of SIRT1 in inhibiting the activity of the NLRP3 inflammasome (122).

In addition, studies have demonstrated that some insulin secretagogues can also prevent the progression of atherosclerosis. To a certain extent, glyburide has an anti-atherosclerotic effect. Glyburide prevents crystal-induced NLRP3 inflammasome activation in DM, and this inhibition appears to be specific for the NLRP3 inflammasome (123).

### Antioxidants

Oxidative stress is a major hallmark of many diseases, including DM and atherosclerotic disease. Numerous recent preclinical reports suggest that the application of antioxidants can scavenge excess ROS and attenuate the inflammatory response by inhibiting the activation of the NLRP3 inflammasome. Several studies have shown that herbal medicines can improve DM-induced vascular damage and have anti-inflammatory effects by inhibiting NLRP3 inflammasome signaling. 6-shogaol, a major ginger derivative, has been reported to exhibit beneficial effects on human arterial smooth muscle cells. 6-shogaol treatment can inhibit ROS production, and subsequent NLRP3 inflammasome activation (124). Furthermore, components of NLRP3 inflammasome are extensively expressed from dysfunctional endothelial cells. Oleanolic acid takes inhibitory effect on NLRP3 through improving endothelial function (125). Salidroside has been shown to ameliorate AGEs-induced endothelial inflammation over a long period by regulating AMPK/NF-κB/NLRP3 signaling (126). Rg1 counteracted ROS-mediated inflammation by upregulating the Nrf2/antioxidant response elements pathway, which contributed to inhibit NLRP3 inflammasome (127). In summary, most of the above-mentioned natural compounds have antioxidant properties. They reduce the damaging effects of ROS by inhibiting NLRP3 inflammasome. These findings provide new insights into the complex relationship between inflammation and oxidation and possible strategies for treating vascular complications of DM.

### NLRP3 Inhibitors

The association of NLRP3 inflammasome with a wide range of diseases has sparked intense scientific interest in the discovery of effective inhibitors of NLRP3 inflammasome. Multiple targets can be used to inhibit NLRP3 inflammasome by exploiting its complex signaling cascade. For example, potential NLRP3 inflammasome inhibition can be targeted by inhibiting NLRP3 inflammasome activation, inhibiting upstream signaling, blocking inflammasome assembly, cycasparase-1 activation inhibition, blocking pore-forming protein Gasdermin D (GSDMD) cleavage, and neutralizing inflammatory cytokines produced by NLRP3 inflammasome. Different mechanisms can be selected to achieve these results, such as inhibition of NLRP3 inflammasome assembly, inhibition of P2X7 receptor, inhibition of K efflux, and ROS scavengers can be used.

MCC950 is an NLRP3 inflammasome-specific inhibitor that inhibits NLRP3 activity by blocking ASC oligomerization and NLRP3 ATP hydrolysis (128). Numerous studies have demonstrated that MCC950 can reduce inflammation, improve vascular function, and prevent DM-related atherosclerosis in streptozotocin-induced ApoE^-/-^ mice (95). Compared to patients with atherosclerosis alone, patients with DM and atherosclerosis have been found to have an approximately four-fold increase in the extent of atherosclerotic lesions, but MCC950 significantly attenuated the lesion size (95). Decreased lesions have been associated with attenuated expression of inflammatory genes such as IL-1β, TNF-α, ICAM-1 and MCP-1. In addition, the vascular function of diabetic blood vessels in mice treated with MCC950 was improved. Studies have also shown that MCC950 treatment attenuated high glucose-induced endothelial cell dysfunction, possibly in part by inhibiting the NEK7-NLRP3 interaction (129). Treatment with MCC950 also attenuated DM-related vascular dysfunction in a mouse model of DM (89). Plasma aldosterone levels in diabetics help increase the expression of inflammatory markers. Nathanne et al. demonstrated the important role of NLRP3 inflammasome activation and its association with aldosterone in diabetic vascular dysfunction. This study confirmed that MCC950 inhibited endothelial dysfunction in DB/DB mice *in vitro* and *in vivo (* 130).

Glyburide is a sulfonylurea drug which is widely used in the United States for the treatment of T2DM. It inhibits ATP-sensitive K^+^ channels in pancreatic β cells. One study conducted by Lamkanfi et al. showed that glyburide prevents PAMPs, DAMPs, and crystal-induced NLRP3 inflammasome activation in bone marrow-derived macrophages (131). They demonstrated that glyburide acts upstream of Cryopyrin and downstream of the P2X7 receptor to block Cryopyrin-dependent inflammasome activation by PAMPs, DAMPs, and crystalline substances. On the basis of glibenclamide, the researchers found a large number of derivatives that also inhibit NLRP3.Examples include 16673-34-0, JC124, and FC11A-2 (132). Hill et al. have reported on the synthesis and biological evaluation of nine sulfonylureas that inhibit NLRP3 activation in mouse bone marrow-derived macrophages in an effective dose-dependent manner. Six of these compounds inhibited NLRP3 at nanomolar concentrations and also stimulated insulin secretion in mouse pancreatic cell lines. These novel compounds have an unprecedented dual mode of action, paving the way for a new generation of sulfonylureas that can be used as therapeutic candidates and/or tool compounds in T2D and its associated inflammatory complications (133).

Neointima hyperplasia is the pathological basis of atherosclerosis and restenosis, which have been associated with diabetes mellitus. Fibroblast growth factor 21 (FGF21) is a potential diabetic drug. Wei et al. found that FGF21 significantly inhibited neointima hyperplasia and improved endothelium-independent contraction in the wire-injured common carotid artery (90) of diabetic mice, which mimics the effects of NLRP3 inflammasome inhibitor MCC950 and caspase 1 inhibitor WEHD.

In addition, Yi et al. demonstrated that inhibition of NLRP3‐dependent inflammation by tranilast is efficient to reverse the metabolic disorders in diabetic mice. Tranilast exhibits anti-vascular inflammatory and anti-atherosclerotic properties by increasing NLRP3 ubiquitination and preventing NLRP3 inflammasome activation (134).

Therefore, NLRP3 inflammasome inhibitors may be the best choice for the treatment of endothelial dysfunction, providing a novel therapeutic strategy to improve DM-related vascular diseases.

## Summary

This study summarizes relevant literature in relation to the major potential of directly or indirectly modulating NLRP3 inflammasome activation in combating diabetic atherosclerotic lesions. We conclude that NLRP3 plays a key role in promoting the formation and development of atherosclerosis in diabetes. In the context of diabetes, risk factors such as high glucose, oxidative stress, and inflammation can induce endothelial dysfunction, inflammation, and platelet activation and aggregation by regulating NLRP3 inflammasome, thereby promoting atherosclerosis.

Inhibiting NLRP3 inflammasome activation have been used to develop therapeutic strategies, such as inhibiting upstream signaling pathways, inhibiting NLRP3 inflammasome activation, blocking NLRP3 inflammasome assembly, and inhibiting caspase-1 activation and the secretion of IL-1β. Antidiabetic drugs, such as hypoglycemic drugs, anti-inflammatory or antioxidant drugs, can improve vascular dysfunction by inhibiting the NLRP3 inflammasome directly or indirectly. These findings improve understanding of the molecular mechanism of NLRP3 inflammasome-associated DM-promoting atherosclerosis and may provide new targets for the development of future treatments.

## Author Contributions

JY, LL, MW, and QM designed the article structure; LL and JY wrote the manuscript; QM and LL drawn the figure; YT, BL, QZ, JL, and BZ helped revise the manuscript. GS and HL were responsible for the supervision and project administration. All authors discussed, edited, and approved the final version.

## Funding

This work was supported by the National Natural Science Foundation of China (Grant no. 81973514) and CAMS Innovation Fund for Medical Sciences (CIFMS) (Grant no. 2021-I2M-1-031).

## Conflict of Interest

The authors declare that the research was conducted in the absence of any commercial or financial relationships that could be construed as a potential conflict of interest.

## Publisher’s Note

All claims expressed in this article are solely those of the authors and do not necessarily represent those of their affiliated organizations, or those of the publisher, the editors and the reviewers. Any product that may be evaluated in this article, or claim that may be made by its manufacturer, is not guaranteed or endorsed by the publisher.
